# Ultrasound Diagnosis of Fetal Neck Masses: A Case Series

**DOI:** 10.1155/2013/243590

**Published:** 2013-01-15

**Authors:** Shakina Rauff, Tan Eng Kien

**Affiliations:** Department of Obstetrics & Gynaecology, National University Hospital, Singapore 119074

## Abstract

Fetal neck masses are rare and may not be apparent during the second trimester fetal anomaly screening scan. It is essential to distinguish the different pathologies as it influences prenatal counseling, antenatal, and postnatal management. Furthermore, some causes may be amenable to in utero treatment. Others have a poor prognosis due to their association with congenital syndromes and aneuploidies. Differentiating the various neck masses and reaching an accurate diagnosis are a challenge. This requires a systematic approach, time and patience, together with an experienced sonographer. Ancillary investigations like karyotyping and magnetic resonance imaging may be used as well. It is important to attain an accurate diagnosis and to follow up the fetus with serial scans as this affects antenatal counselling and prognosis as well as the mode of delivery. Here, we present four cases of fetal neck masses that were seen at our antenatal diagnostic centre and highlight the distinguishing ultrasound features of each. This will enable one to approach the ultrasound diagnosis of fetal neck masses in a methodical and logical manner.

## 1. Introduction

The ultrasound is the modality of choice for prenatal screening of fetal anomalies. Over the years, advances in ultrasonography have enabled better elucidation of anomalies of the central nervous and cardiovascular systems, abdomen, and limbs. However, fetal neck masses are rare and may be overlooked. Accurate antenatal diagnosis of neck pathology remains a challenge. Distinguishing the different neck masses is important for appropriate antenatal counselling of prognosis, mode of delivery, and postnatal management, all of which have to be planned. Some pathologies, for example, cystic hygroma are associated with chromosomal aneuploidies and nonchromosomal syndromes. Others like lymphangiomas may be amenable to in utero intervention [1].

Congenital tumours of the cervical region can be subdivided into anterior and posterior masses. The commonest fetal neck mass is cystic hygroma [2] and the commonest fetal neck tumour is cervical teratoma. Varying ultrasound features and supplementary investigations, for example, karyotype aid in further diagnosis.

Here we present 4 rarer cases of fetal neck masses that were seen at our antenatal diagnostic centre over the last year (2009-2010) and a diagnostic approach to them.

## 2. Case 1: Clear Cystic Mass (Bronchogenic Cyst)

**Figure 1:**
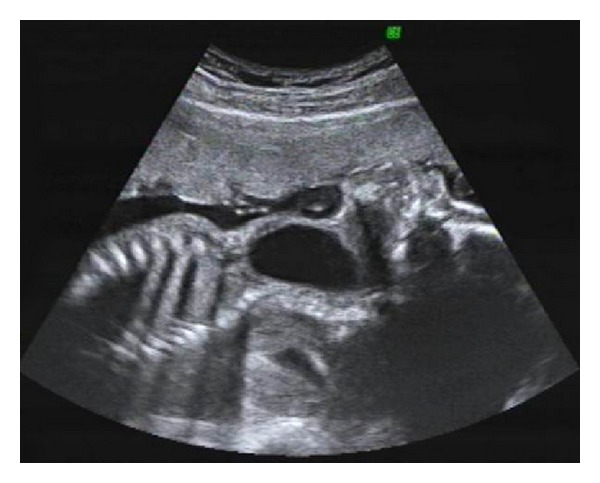
Ultrasound image at 27 weeks' gestation of a bronchogenic cyst.

A 32-year-old primigravida was referred to our unit at 27 weeks' gestation for a neck mass and polyhydramnios. The mass, first detected at 15 weeks' gestation, was reported as a 3 cm clear cystic mass on the anterior neck. A cystic hygroma was suspected. Amniocentesis performed then showed a normal male karyotype. At our unit, we found a clear, nonseptated 4 cm mass in the anterior neck with hydramnios (Figure 1), the amniotic fluid index being 29 cm. The stomach also appeared small. No other fetal anomalies were present. Our ultrasound diagnosis was a branchial cyst. Serial scans showed a stable cyst with no worsening of polyhydramnios. She delivered at term via elective Caesarean section. Postnatal excision revealed a bronchogenic cyst.

## 3. Case 2: Solid-Cystic Mass (Cervical Teratoma)

**Figure 2:**
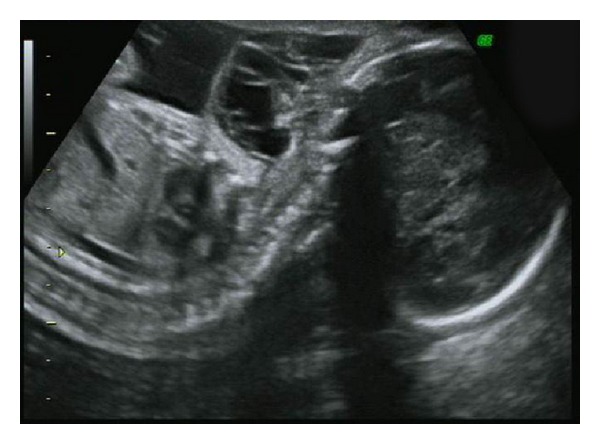
Ultrasound image at 26 weeks' gestation of a cervical teratoma.

The second case was also referred to our centre at 26 weeks' gestation when a neck mass was detected incidentally on ultrasound. Her antenatal course had been hitherto uneventful. Our scan found a 5 cm multiloculated anterior neck mass between the lower jaw and upper chest. The mass had mixed solid-cystic areas and calcifications. There were well-defined borders and no increased vascularity (Figure 2). An epignathus was excluded as the tumour was not arising from the mouth. The amniotic fluid index was normal at 14 cm. The diagnosis then was a cervical teratoma. The patient delivered via elective Caesarean section at term. A teratoma was confirmed postnatally after surgical excision.

## 4. Case 3: Solid Vascular Mass (Haemangioma)

**Figure 3:**
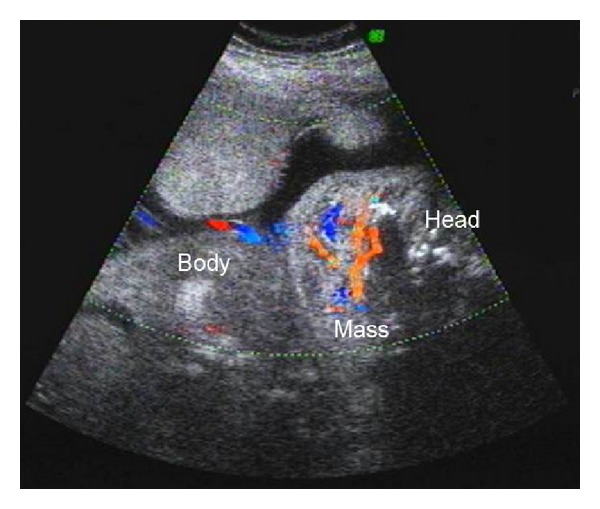
Ultrasound image at 29 weeks' gestation of a vascular neck mass—likely haemangioma.

This patient was admitted with symptoms of preterm labour at 29 weeks'. A scan performed showed all the features of fetal hydrops, namely, ascites, pleural, and pericardial effusions. A large, highly vascular anterolateral neck mass with variable echogenicity and mainly solid areas was also discovered. The amniotic fluid index was 17 cm. Thus our sonographic diagnosis was hydrops secondary to high output cardiac failure due to a vascular neck mass—probably a haemangioma. The patient delivered a macerated stillborn male baby at 30 weeks' gestation. Clinically the baby had a large purplish swelling over the neck consistent with a haemangioma (Figure 3). A postmortem examination was declined.

## 5. Case 4: Solid Avascular Mass (Lymphangioma)

**Figure 4:**
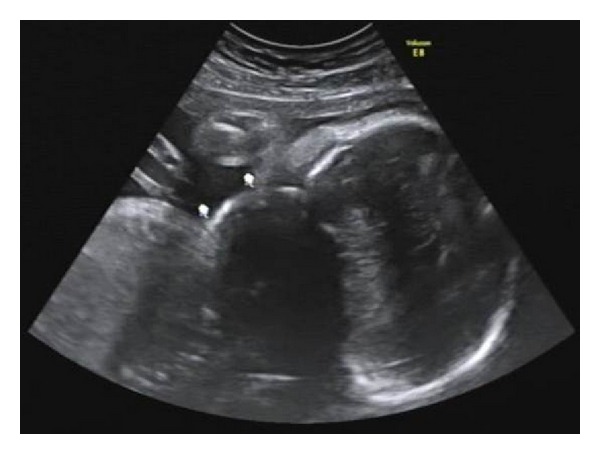
Ultrasound image at 30 weeks' gestation of a lymphangioma.

The patient was a 33-year-old who was detected to have a right-sided, unilateral 5 cm neck mass at a 30-week scan. This extended posteriorly up to the cervical spine. The mass had thin walls with solid areas present and few echogenic areas (Figure 4). Vascularity was not increased. The amniotic fluid index was 11 cm. Our provisional diagnosis was a possible teratoma, lymphangioma, or neuroblastoma as the location and nature of the mass made the diagnosis of a thyroid lesion or cystic hygroma unlikely. The mass was stable with no development of hydramnios in subsequent scans. The patient underwent an elective Caesarean section at term. The mass was excised postnatally and histologically it was a lymphangioma.

## 6. Discussion

All the masses except for one were diagnosed in the late second and early third trimester. This may be because they were not looked for during the routine fetal anomaly scan or development occurred in the later part of the pregnancy. One should look out for them routinely during the fetal anomaly scan and again if another scan is done in the third trimester or if the patient presents with polyhydramnios.

A first step in aiding this diagnosis is the subdivision of the neck mass by their anatomic location into anterior and posterior masses. The commoner differentials of an anterior neck mass include teratoma, epignathus, goiter, and haemangioma and those of a posterior cervical mass include cystic hygroma, cervical meningocele, occipital encephalocele, haemangioma, and lymphangioma.

Scanning systematically for distinguishing features should be carried out once the mass is localised as anterior or posterior. The differentiating features that should be noted include the solid or cystic nature of the mass, the thickness of its wall, the vascularity, presence of calcifications and septations or loculations and the laterality, that is, unilateral or bilateral of the mass. In all cases, it is essential to examine for the amniotic fluid volume, other fetal anomalies, the presence of hydrops, and the fetal neck position.

In the case of the fetus with the cervical teratoma, one would find an anterior, solid-cystic neck mass with no increase in vascularity. Cervical teratomas are unilateral, multiloculated and may have calcifications. They are asymmetrical and are associated with hydramnios. The other differential diagnosis for an anterior neck mass would be a goitre which has a uniformly solid appearance without septations. Goitres are usually bilateral, symmetrical, and in the midline.

One of the commonest posterior neck masses is a cystic hygroma and its features are easily distinguishable—it is a cystic mass with thin walls and septations. Calcifications and vascularity are absent. Cystic hygromas are bilateral, usually detected in the first trimester and are associated with Turner's syndrome and hydrops. Our last patient had a fetus with a lymphangioma. This posterior neck mass has a solid-cystic appearance with thin walls and minimal vascularity. It is a unilateral, multiloculated mass with poorly-differentiated borders. Occipital encephaloceles, however, have thick walls and no loculations although they are solid-cystic in nature as well. They are found in the midline and a calvarial defect may be present.

Haemangiomas may be present as an anterior or posterior neck mass. In our third patient, the haemangioma was found to be anterolateral with a solid-cystic appearance and thick walls. The mass is highly vascular and may have variable echogenicity.

Ancillary investigations like magnetic resonance imaging (MRI) can be useful in the differential diagnosis of a fetal neck mass, especially when ultrasound is inconclusive or difficult, for example, in the obese patient, unfavourable fetal position. Although MRI is principally helpful in evaluating anomalies of the central nervous system, it may be valuable in assessing the degree of airway compression caused by the neck tumour, especially solid anterior ones. This cannot be estimated as accurately with ultrasound. MRI does not involve ionizing radiation, is good for soft tissue differentiation, and it is being increasingly utilised as an adjunct to ultrasound in prenatal diagnosis [3]. However, we did not use MRI to gauge the tracheal compression in our cases.

In conclusion, an experienced and meticulous sonographer is required for the prenatal diagnosis of fetal neck masses. A thorough and systematic examination of the fetus is also necessary due to the association with other malformations. This, together with supplementary investigations like karyotyping and MRI will enable the obstetrician to adequately counsel the patient about the prognosis of the baby and the mode of delivery. Lastly, a multidisciplinary approach involving the obstetrician, neonatologist, and paediatric surgeon is crucial to ensure the best outcome possible.
